# Synergistic effects of tanshinone IIA and mesenchymal stem cells on neuroprotection, cardiovascular repair, tissue regeneration, muscle protein synthesis, and gut microbiota modulation across neurodegenerative, cardiovascular, and regenerative disease models

**DOI:** 10.3389/fcell.2026.1883952

**Published:** 2026-08-26

**Authors:** Wenzhe Wang, Hongyan Gao, Yuan Wang

**Affiliations:** 1 School of Medicine, Pingdingshan University, Pingdingshan, Henan, China; 2 Internal Medicine, Brookdale University Hospital Medical Center, New York, NY, United States

**Keywords:** exosomes, inflammation, mesenchymal stem cells, miR-223-5p, Nrf2 pathway, oxidative stress, SDF-1/CXCR4 axis, synergistic therapy

## Abstract

Neurodegenerative, cardiovascular, and regenerative disorders remain major causes of disability and mortality worldwide, necessitating the development of multimodal therapeutic strategies. Tanshinone IIA (Tan IIA), a bioactive diterpenoid isolated from Salvia miltiorrhiza, possesses potent antioxidant, anti-inflammatory, anti-apoptotic, and pro-angiogenic properties. Mesenchymal stem cells (MSCs) have emerged as promising regenerative therapeutics owing to their differentiation potential, immunomodulatory capacity, and paracrine effects. Growing evidence indicates that the combination of Tan IIA and MSCs exerts synergistic therapeutic effects by enhancing MSC survival, proliferation, migration, and regenerative function while mitigating oxidative stress and inflammatory injury. This review summarizes current evidence regarding the combined effects of Tan IIA and MSCs on neuroprotection, cardiovascular repair, tissue regeneration, muscle protein synthesis, and gut microbiota modulation across neurodegenerative, cardiovascular, and regenerative disease models. The underlying molecular mechanisms involve the regulation of PI3K/Akt, Wnt/β-catenin, TGF-β/Smad, and Nrf2 signaling pathways, together with improvements in angiogenesis, immune homeostasis, mitochondrial function, and cellular metabolism. Emerging evidence also suggests that modulation of the gut microbiota may contribute to enhanced systemic immune regulation and regenerative outcomes, whereas improvements in muscle protein synthesis may support tissue recovery and functional restoration. Although preclinical findings are promising, further mechanistic investigations and well-designed clinical trials are required to optimize treatment protocols and establish the efficacy, safety, and translational potential of this combinational therapeutic strategy.

## Introduction

Mesenchymal stem cells (MSCs) have emerged as a cornerstone of regenerative medicine due to their multipotent differentiation capacity, paracrine signaling, and immunomodulatory functions (Santoro et al., 2025; Wong et al., 2026). They have been widely investigated in the treatment of neurological, cardiovascular, musculoskeletal, and fibrotic diseases (Wong et al., 2026). Despite encouraging preclinical outcomes, MSC-based therapies face significant challenges, including poor survival after transplantation, limited homing and engraftment efficiency, and impaired regenerative function in hostile microenvironments characterized by inflammation, oxidative stress, and apoptosis (Wong et al., 2026; Tu et al., 2026). These limitations underscore the need for strategies that can enhance MSC viability and therapeutic efficacy.

Tanshinone IIA (TIIA), a lipophilic diterpene quinone extracted from Salvia miltiorrhiza (Danshen), has long been used in traditional Chinese medicine and is now recognized for its broad pharmacological properties (Hu et al., 2025; Jiang et al., 2019). TIIA exhibits anti-inflammatory, antioxidative, anti-apoptotic, and pro-angiogenic effects across multiple disease contexts (Hu et al., 2025; Du et al., 2025). Increasing evidence suggests that TIIA not only confers direct protective benefits but also modulates the biological functions of stem cells, enhancing their therapeutic performance (Han et al., 2025).

Recent studies have demonstrated that combining TIIA with MSCs, either through preconditioning, incubation, exosome modification, or co-administration, produces synergistic outcomes in diverse pathological models (Song et al., 2025). In neurological diseases such as AD, VaD, SCI, and ischemic stroke, TIIA-modified MSCs significantly improve cognitive recovery, attenuate neuroinflammation, and promote neuronal differentiation compared to MSCs alone (Table 1). In cardiovascular injury models, particularly myocardial infarction (MI) and ischemia/reperfusion injury, TIIA-pretreated MSCs or their exosomes enhance cardiac repair by reducing apoptosis, suppressing inflammatory pathways, and promoting angiogenesis (Shen et al., 2025). Furthermore, in osteoporotic fracture healing (Li Y. et al., 2024), liver cirrhosis (Yi et al., 2025), and intrauterine adhesion (Xu W. et al., 2022), TIIA supports stem cell survival, preserves differentiation potential, and mitigates fibrosis.

**TABLE 1:** Summary of preclinical studies investigating Tanshinone IIA (TIIA) and mesenchymal stem cell (MSC)-based therapies across different disease models.

Study	Disease model (animal/Cell)	MSC source	TIIA formulation and dose	Treatment strategy	Route and duration	Major mechanisms	Main outcomes
Huang et al. (2019)	Amyloid-β_25_–_35_-induced Alzheimer’s disease rat model	Bone marrow MSCs	TIIA preconditioning	Intracerebroventricular transplantation of TIIA-preconditioned MSCs	Intracerebroventricular injection following Aβ_25_–_35_ induction	Anti-inflammatory; ↓BACE1, ↓PS1; inhibition of IL-1β, IL-6, TNF-α	Improved Morris water maze performance; reduced neuroinflammation; enhanced neuronal survival compared with MSCs alone
Ba et al. (2022)	Amyloid-β_(25_–_35)_-induced Alzheimer’s disease rat model	Bone marrow MSCs	TIIA preconditioning	Intrahippocampal transplantation of TIIA-preconditioned MSCs	Intrahippocampal injection after Aβ_25_–_35_ induction	AMPK activation; antioxidant signaling; reduced Tau phosphorylation	Improved cognition, reduced oxidative stress, decreased Aβ deposition and Tau hyperphosphorylation
Kong et al. (2017)	LPS-induced neuroinflammation (mice)	Bone marrow MSCs	TIIA-preconditioned MSCs	TIIA-preconditioned MSC Transplantation	MSC transplantation in LPS-treated mice	Enhanced immunomodulation	Greater reduction of IL-1β, IL-6, TNF-α; improved Y-maze and Morris water maze performance
Kong et al. (2017)	Rat vascular dementia (VaD) induced by modified four-vessel occlusion (4-VO)	Bone marrow MSCs	TIIA co-administration	Combined TIIA treatment with BMSC transplantation	Tail vein intravenous injection	Anti-apoptosis; anti-Tau; cholinergic protection	Improved learning and memory; reduced neuronal apoptosis; enhanced cholinergic activity
Li Y. et al. (2024)	Rat spinal cord injury (SCI)	Bone marrow MSCs	TIIA, 30 mg/kg (tail vein, 1 h before SCI), followed by 20 mg/kg/day for 7 days	Combined TIIA treatment with BMSC transplantation	Intravenous TIIA; intraspinal transplantation of 1 × 10^7^ PKH67-labeled BMSCs	Promoted neuronal differentiation	Increased BBB locomotor scores; ↑NeuN, Nestin, NF200; ↓GFAP; reduced lesion cavity
Shen et al. (2025)	Mouse spinal cord injury	Umbilical cord MSC-derived exosomes	TIIA -pretreated UCMSC-derived exosomes (T-Exos)	UCMSCs were preconditioned with TIIA to generate therapeutic exosomes	Exosome administration following SCI (*in vivo*)	miR-223-5p/USP8/NLRP3; M2 polarization	Improved motor recovery; reduced IL-1β and IL-18; suppressed NLRP3 inflammasome
Huang et al. (2022)	LPS-treated N9 microglia	Bone marrow MSCs	TIIA 10 μM for 48 h (MSC preconditioning)	TIIA-incubated MSCs (TIIA-MSCs) co-cultured with LPS-stimulated N9 microglial cells using a Transwell system	MSCs pretreated with TIIA for 48 h before indirect co-culture; LPS (1 μg/mL, 24 h) used to induce inflammation	TREM2 signaling	Stronger suppression of IL-6 and TNF-α; TREM2 knockdown abolished benefit
Lu et al. (2016)	Rat acute cerebral infarction	Bone marrow MSCs	TIIA	Combination of TIIA administration with BMSC transplantation	BMSC transplantation with concurrent TIIA treatment after cerebral infarction	↑BDNF; ↓Nogo-A/NgR	Improved Longa neurological scores and reduced apoptosis
Kaiser et al. (2022)	Pig ischemic stroke	Induced pluripotent stem cell-derived neural stem cells (iNSCs)	TIIA-loaded nanoparticles	Pretreatment with Tan IIA-NPs combined with iNSC transplantation	Intracisternal administration; evaluated over 12 weeks	Enhanced cell survival	Reduced infarct volume and improved neurological recovery
Jeon et al. (2023)	Pig ischemic stroke model	Induced pluripotent stem cell-derived neural stem cells (iNSCs)	TIIA-loaded nanoparticles	Combination therapy using Tan IIA-NPs and iNSC transplantation	Intracisternal administration; evaluated up to 12 weeks post-treatment	Gut-brain axis regulation	Reduced intestinal inflammation, improved neurological recovery
Shen et al. (2025)	Rat myocardial ischemia/reperfusion injury (MI/RI) model	Mesenchymal stem cell-derived exosomes (MSC-exo)	TIIA (TSA) pretreatment of MSCs before exosome isolation	Cell-free therapy using TSA-preconditioned MSC-derived exosomes (TSA-MSCexo)	Administration of purified TSA-MSCexo following MI/RI induction	miR-223-5p/CCR2	Reduced infarct size; enhanced angiogenesis; improved cardiac function
Tong et al. (2011)	Rat myocardial ischemia model and *in vitro* BMSC migration assay	Bone marrow-derived mesenchymal stem cells (BMSCs)	TIIA (2 μM *in vitro*; *in vivo* administration)	Combined TIIA treatment with systemic BMSC transplantation	Systemic BMSC transplantation with concurrent TIIA treatment after MI induction	SDF-1/CXCR4	Increased MSC homing; infarct size reduced from 46.95% to 31.46%; improved cardiac function
Li Y. et al. (2024)	*In vitro*: H_2_O_2_-induced oxidative stress in MSCs; *In vivo*: ovariectomized mouse tibial fracture model	Mouse mesenchymal stem cells	TIIA pretreatment (*in vitro*; concentration-dependent) and local TIIA-loaded injectable biodegradable hydrogel (*in vivo*)	Pharmacological protection of MSCs combined with local sustained TIIA delivery to fracture site	MSC pretreatment before H_2_O_2_ exposure; local hydrogel implantation at fracture site; fracture healing assessed after 4 weeks	Nrf2 activation	Increased bone mineral density, callus formation and biomechanical strength; reduced apoptosis
Lu et al. (2016)	Primary MuSC culture and mouse skeletal muscle injury model	Primary skeletal muscle stem cells (MuSCs)	TIIA (*in vitro*; dose-dependent)	Direct pharmacological treatment of MuSCs	Cell culture and mouse muscle injury model	MAPK/Akt	Enhanced MuSC proliferation, differentiation and muscle regeneration
Yi et al. (2025)	Liver cirrhosis induced by CCl_4_ plus 10% alcohol in Sprague–Dawley rats	Endogenous hepatic stem/progenitor cells (EpCAM^+^/OV-6^+^)	TIIA 10, 20, or 40 mg/kg	TIIA monotherapy	Intraperitoneal injection for 7 consecutive days	Stem cell activation; anti-fibrosis	Increased EpCAM^+^/OV-6^+^ progenitors; improved liver function; reduced fibrosis
Gao et al. (2020)	*In vitro*: RAW264.7 macrophages, bone marrow MSCs, HUVECs. *In vivo*: Rat subcutaneous implantation model	Endogenous MSC recruitment	TIIA -loaded aligned microfiber scaffold (AF-1) containing 1 μM TIIA (sustained release)	Scaffold-based delivery	Local implantation of aligned microfiber scaffold; *in vitro* conditioned-medium assays and *in vivo* subcutaneous implantation	M2 macrophage polarization	Enhanced MSC recruitment, angiogenesis and tissue regeneration
Xu W. et al. (2022)	Intrauterine adhesion (IUA) model in female Sprague–Dawley rats (4–6 weeks)	First-trimester human decidual MSCs (dMSCs)	TIIA solution, 11 mg/kg/day	TIIA alone, dMSCs alone, or combined TIIA + dMSCs	TIIA: intragastric administration for 7 days dMSCs: parauterine injection of 0.2 mL (1 × 10^6^ cells/mL	miR-29a/TGF-β1/Smad3	Reduced fibrosis; increased gland number and endometrial thickness; restored E2 levels

However, despite this growing body of evidence, several key scientific gaps remain. Most studies are restricted to preclinical animal models, with a lack of human clinical data validating the safety and efficacy of TIIA-modified MSC therapies. The molecular mechanisms underlying the enhanced therapeutic effects are not fully elucidated, with pathways such as Nrf2, TREM2, SDF-1/CXCR4, and miR-223-5p only partially characterized. MSCs have attracted considerable attention for their broad clinical potential, encompassing not only tissue regeneration and wound repair but also significant immunomodulatory functions. Moreover, the optimal dosage, timing, and administration strategies for combining TIIA with MSCs remain poorly defined. Finally, it is unclear whether the observed benefits are sustained long-term or primarily reflect short-term enhancement of MSC survival and function. Addressing these gaps is crucial for translating the promising synergy between TIIA and MSCs into clinically viable regenerative therapies.

### Search strategy

A comprehensive literature search was performed in PubMed, Web of Science, Scopus, and Embase from database inception to August 2025 to identify studies investigating the therapeutic effects of TIIA in combination with MSCs or stem cell–derived products. The search strategy used both Medical Subject Headings (MeSH) and free-text terms, including “Tanshinone IIA,” “Tanshinone II,” “TIIA,” “Danshen,” “Salvia miltiorrhiza,” combined with “mesenchymal stem cell,” “MSC,” “bone marrow stromal cell,” “neural stem cell,” “induced pluripotent stem cell,” “stem cell–derived exosome,” and “extracellular vesicle.” Only original experimental studies, preclinical animal studies, and *in vitro* mechanistic investigations published in English were included. Additional relevant articles were identified through manual screening of reference lists. Eligible studies were required to assess TIIA either alone or in combination with MSCs/exosomes in disease models relevant to neurology, cardiology, orthopedics, hepatology, or reproductive medicine. Studies were excluded if they did not involve MSCs or related products, if they were reviews, case reports, or editorials, or if they were published in languages other than English. Data extracted from eligible publications included author, year, source of TIIA, type of stem cell intervention, disease model, delivery strategy, and reported therapeutic outcomes.

### Mesenchymal stem cells

MSCs have attracted considerable attention for their broad clinical potential, encompassing not only tissue regeneration and wound repair but also significant immunomodulatory functions (Pînzariu et al., 2025). MSCs are multipotent, self-renewing stem cells with high proliferative capacity, and they can be isolated from a wide range of tissues, including bone marrow, adipose tissue, placenta, umbilical cord, skin, skeletal muscle, liver, and spleen (Han et al., 2025; Orbay et al., 2012). The defining feature of MSCs is their ability to differentiate into a broad spectrum of lineages, including chondrogenic, osteogenic, myogenic cells, adipogenic, and neuronal (Almalki and Agrawal, 2016; Huang et al., 2011). MSCs naturally possess the ability to differentiate into multiple cell types, such as osteoblasts, chondrocytes, adipocytes, as well as neuronal and muscle cells (Meirelles Lda and Nardi, 2009; Xu et al., 2024). Coupled with their ease of *in vitro* expansion and minimal ethical concerns, these properties have made MSCs highly appealing for developing therapeutic strategies for a wide range of human diseases. Studies have highlighted the role of MSCs in tissue regeneration, owing to their capacity to home to injury sites, secrete growth factors and cytokines, and exert paracrine effects that support repair (Pînzariu et al., 2025; Zhan et al., 2020). Research has shown that MSCs play a key role in tissue regeneration due to their ability to migrate to injury sites, their paracrine activity, secretion of growth factors and cytokines, and low immunogenicity, which enables allogeneic transplantation without the need for immunosuppressive therapy (Ma et al., 2020; Dabrowska et al., 2020). Following transplantation, MSCs acquire immunosuppressive capabilities, integrate into the architecture of the host tissue, and differentiate into progenitor cells within the microenvironment, thereby contributing both as stem cells and through their progeny (Pînzariu et al., 2025; Ding et al., 2010). MSCs have an intrinsic ability to sense alterations in their surroundings, such as the onset of inflammation, and in response, they can release bioactive factors and generate progenitor cells to adapt to these changes (Han et al., 2022). Furthermore, studies have shown that MSCs are capable of migrating to distant sites of inflammation beyond their initial injection location (Glenn and Whartenby, 2014). As research in regenerative medicine and cell-based therapies advances, MSCs are increasingly acknowledged for their significant therapeutic potential. Their diverse properties often act synergistically to achieve desired biological outcomes. Nevertheless, the precise mechanisms through which MSCs exert these therapeutic effects remain incompletely understood.

In clinical practice, the therapeutic potential of MSCs is being actively explored in trials involving patients with a wide range of conditions, including orthopedic injuries, acute graft rejection after bone marrow transplantation, cardiovascular diseases, autoimmune disorders, and liver disorders (Pînzariu et al., 2025). Additionally, genetic modification of MSCs to overexpress antitumor genes has created promising opportunities for their use as a novel antineoplastic therapy (Garza Treviño et al., 2024). Overall, MSCs demonstrate a favorable safety profile, with most studies reporting no significant medium-term adverse effects. A few investigations have noted mild, transient reactions around the injection site, but these effects are generally short-lived and not clinically concerning.

### MSC source heterogeneity and TIIA pretreatment strategies

A key contributor to heterogeneity across the included studies is the variation in the diversity of MSC origins, TIIA pretreatment protocols, and delivery strategies. MSCs obtained from bone marrow (BM-MSCs), umbilical cord (UC-MSCs), adipose tissue (AD-MSCs), and decidua (dMSCs) have distinct biological properties that may affect their responsiveness to TIIA.

BM-MSCs have been the most commonly studied source, largely due to their strong osteogenic and neuroregenerative potential, and they were used in studies addressing myocardial infarction, spinal cord injury, vascular dementia, and fracture healing (Cheng et al., 2024; Ardah et al., 2026; Shu et al., 2016; Li Y. et al., 2024). Compared with adult MSCs, UC-MSCs show greater proliferative capacity, lower immunogenicity, and stronger immunomodulatory activity, which makes them well suited to exosome-based therapies (Kong et al., 2017). Consistent with this, the recent SCI study by Shen and colleagues reported that exosomes generated from TIIA-preconditioned UC-MSCs exerted marked anti-inflammatory effects via enrichment of miR-223-5p (Shen et al., 2025). In reproductive regeneration, decidual MSCs were chosen because of their inherent ability to adapt to the uterine microenvironment, where they worked synergistically with TIIA to support endometrial repair (Xu W. et al., 2022). Although adipose-derived MSCs have excellent proliferative capacity and are clinically accessible, direct evidence about their interaction with TIIA is still limited.

Substantial variability is also evident in TIIA administration protocols. In most *in vitro* experiments, MSCs underwent short-term pretreatment prior to transplantation, whereas *in vivo* studies generally delivered TIIA systemically or locally after cell transplantation. Pretreatment length ranged from several hours to 48 h depending on the disease model, yet no standardized protocol has been established. Similarly, TIIA doses varied considerably across studies based on experimental design and the delivery route, which prevents quantitative comparison of dose–response relationships.

Delivery approaches likewise differed across studies. Some investigators transplanted TIIA-preconditioned MSCs directly, while others used exosomes isolated from preconditioned MSCs or combined TIIA with biomaterial-based systems such as injectable hydrogels or aligned microfibers (Cheng et al., 2024; Huang et al., 2019; Li Y. et al., 2024). Hydrogel-assisted delivery is particularly promising, as it can both extend local TIIA release, increase MSC retention, and enhance tissue regeneration. Nonetheless, the lack of standardized pretreatment conditions, MSC sources, and administration regimens remains a major barrier to comparing outcomes across studies and should be addressed as a priority in future work.

## MSCs targets and treatment consequences

### Enhancing stem cell efficacy through pharmacological modulation

Stem cell–based therapies represent a promising strategy in regenerative medicine; however, their clinical translation remains limited by several biological challenges, including poor post-transplantation survival, insufficient homing to injured tissues, and functional impairment within hostile pathological microenvironments characterized by oxidative stress, inflammation, and hypoxia. To address these limitations, increasing attention has been directed toward pharmacological modulation, whereby bioactive compounds are used to prime, precondition, or synergize with stem cells to enhance their therapeutic efficacy. This strategy aims to improve not only stem cell viability and differentiation capacity but also their paracrine signaling activity and the regenerative potential of stem cell–derived extracellular vesicles.

**FIGURE 1:**
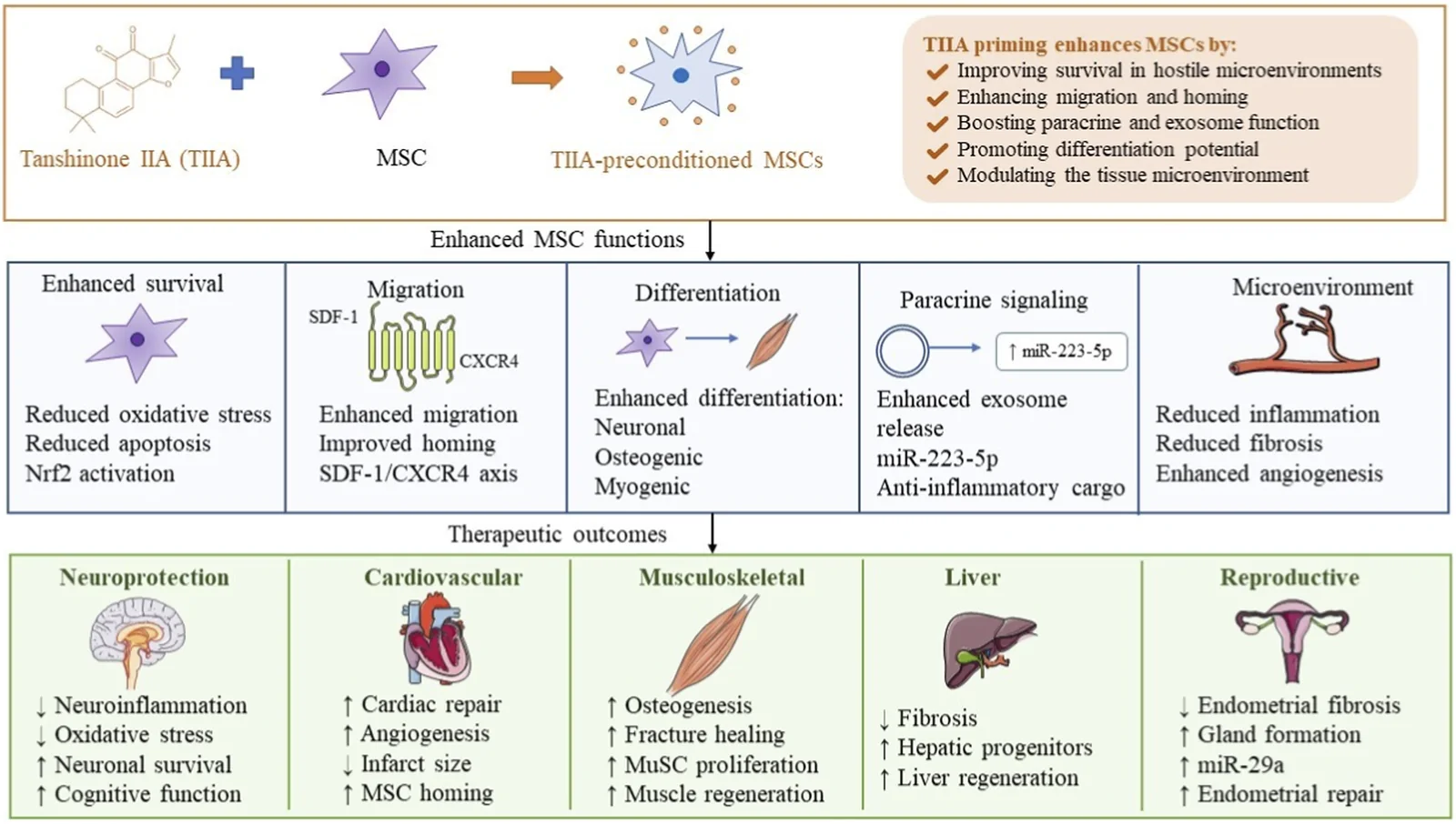
Mechanistic overview of the synergistic effects of Tanshinone IIA (TIIA) and mesenchymal stem cells (MSCs) in regenerative medicine. TIIA enhances the therapeutic efficacy of MSCs by improving cell survival, migration, and paracrine activity within damaged tissues. Mechanistically, TIIA activates key protective signaling pathways, including Nrf2, MAPK, Akt, and the SDF-1/CXCR4 axis, which reduce oxidative stress, suppress inflammation, and promote stem cell homing. In addition, TIIA preconditioning modifies the cargo of MSC-derived exosomes, enriching regulatory microRNAs (e.g., miR-223-5p) that inhibit inflammatory signaling pathways such as CCR2-mediated immune cell recruitment. These combined effects enhance angiogenesis, attenuate fibrosis, and support tissue repair across multiple disease models, including cardiovascular, neurological, musculoskeletal, hepatic, and reproductive disorders.

TIIA, a lipophilic diterpene quinone extracted from Salvia miltiorrhiza, has emerged as a promising pharmacological modulator in this context (Hu et al., 2025). TIIA is widely recognized for its anti-inflammatory, antioxidative, anti-apoptotic, and pro-angiogenic properties (Hu et al., 2025; Li H-Z. et al., 2024). Importantly, accumulating evidence suggests that TIIA can enhance the biological functions of MSCs through multiple mechanisms (Figure 1). Preconditioning MSCs with TIIA improves their resistance to oxidative stress, enhances survival in ischemic or inflammatory microenvironments, and modulates key signaling pathways including Nrf2, MAPK, Akt, and stromal cell–derived factor-1 (SDF-1)/CXCR4 (Saparov et al., 2016; Kahrizi et al., 2023). Furthermore, TIIA-treated MSCs and their exosomes have demonstrated enhanced therapeutic efficacy in experimental models of neurodegeneration, cardiovascular injury, bone fracture healing, liver fibrosis, and reproductive disorders, as illustrated in Figure 1.

### Neurodegenerative disease models

MSCs possess intrinsic neuroprotective and immunomodulatory capabilities, which have been widely investigated for their potential in treating neurodegenerative diseases. These cells can secrete a variety of neurotrophic factors, modulate microglial activity, and provide trophic support to injured neurons. TIIA, a bioactive diterpene quinone derived from Salvia miltiorrhiza, exhibits strong anti-inflammatory, antioxidant, and anti-apoptotic properties. When examined individually, both MSCs and TIIA demonstrate distinct but complementary mechanisms of neuroprotection. However, their combined application as TIIA-preconditioned MSCs (TIIA-MSCs) has revealed synergistic effects that substantially enhance therapeutic outcomes, offering insights into novel regenerative strategies for neurodegenerative conditions.

In Alzheimer’s disease (AD) models, TIIA alone provides modest neuroprotective benefits. Studies have shown that TIIA can reduce oxidative stress and suppress neuroinflammatory pathways, leading to limited attenuation of neuronal injury. Nevertheless, its effects on cognitive deficits are restricted, as TIIA cannot replace the regenerative potential or paracrine signaling of MSCs (Huang et al., 2019). MSCs alone can improve cognitive function, attenuate neuroinflammation, and support neuronal survival through the secretion of neurotrophic factors and modulation of microglial activity. However, their therapeutic efficacy is often limited by *in vitro* expansion-induced senescence, reduced proliferation, and diminished paracrine activity (Li Y. et al., 2024). When MSCs are preconditioned with TIIA, their therapeutic potential is significantly amplified. Behavioral studies in Aβ25-35-induced rats demonstrate that TIIA-MSCs provide more pronounced improvements in learning and memory compared to either treatment alone. Mechanistically, TIIA-MSCs synergistically suppress the expression of pro-inflammatory cytokines such as IL-1, IL-4, IL-6, IL-10, and TNF-α, inhibit BACE1 and PS1 to reduce amyloid-β accumulation, and enhance neuronal survival. This combination surpasses the additive effects of TIIA or MSCs alone, providing a potent neuroprotective strategy in AD models (Huang et al., 2019).

In lipopolysaccharide (LPS)-induced neuroinflammation models, the effects of TIIA alone have not been extensively tested, but it is known to attenuate systemic inflammation and oxidative stress. MSCs alone can reduce microglial activation, downregulate IL-1, IL-6, and TNF-α, and moderately improve cognitive function, although their effects are often limited in the context of severe inflammatory challenges (Wu et al., 2024). In contrast, TIIA-modified MSCs exhibit enhanced anti-inflammatory effects, significantly reducing microglial activation and the expression of pro-inflammatory cytokines, while markedly improving spatial learning and memory. These findings indicate that TIIA acts as a pharmacological enhancer, increasing MSC resilience to inflammatory stress and potentiating their paracrine neuroprotective signaling, thereby producing synergistic benefits that exceed the effects of either treatment alone (Wu et al., 2024).

Vascular dementia (VaD) models further illustrate the differential and combined effects of TIIA and MSCs. TIIA alone partially reduces neuronal apoptosis and modestly improves cognitive performance by attenuating oxidative stress and tau hyperphosphorylation, yet these effects are insufficient for full functional recovery (Kong et al., 2017). MSCs alone can improve spatial learning and memory, reduce apoptosis, and partially restore cholinergic function; however, their therapeutic efficacy is constrained by limited survival and insufficient paracrine support in ischemic tissue. When combined, TIIA-MSC therapy synergistically enhances MSC survival, further decreases hippocampal apoptosis, reduces tau phosphorylation, and restores cholinergic activity more effectively than either intervention alone. The presence of TIIA primes MSCs to withstand ischemic injury and secrete higher levels of neurotrophic factors, leading to superior cognitive and functional recovery (Kong et al., 2017).

Mechanistically, the synergistic effects of TIIA-MSCs arise from several interconnected pathways. While TIIA alone can moderately suppress pro-inflammatory cytokines, preconditioning MSCs with TIIA amplifies paracrine-mediated cytokine suppression, particularly IL-1, IL-6, and TNF-α, in injured hippocampal tissue. MSCs alone provide partial protection against neuronal apoptosis, but TIIA enhances anti-apoptotic signaling, resulting in greater neuronal survival. Additionally, TIIA-MSCs exert complementary effects on proteinopathy by combining the anti-amyloid and anti-tau properties of TIIA with MSC-mediated trophic support, effectively reducing amyloid-β accumulation and tau hyperphosphorylation. These molecular changes are accompanied by significant improvements in learning and memory, demonstrating that the combination therapy leverages both MSC regenerative capacity and TIIA’s neuroprotective properties to achieve robust cognitive restoration.

In summary, across various neurodegenerative models, TIIA alone confers moderate neuroprotection and anti-inflammatory effects, whereas MSCs alone provide support for neuronal survival and paracrine signaling. Importantly, TIIA-MSC combination therapy consistently exhibits superior efficacy, mediated by enhanced survival, augmented anti-inflammatory and anti-apoptotic signaling, and modulation of disease-specific pathogenic proteins. This synergy underscores the therapeutic promise of pharmacologically optimized MSCs and supports their potential future application in the treatment of AD, VaD, and other neurodegenerative disorders.

### Spinal cord injury and neurological repair

SCI remains a major clinical challenge due to the cascade of secondary damage that follows the initial trauma, including neuroinflammation, excitotoxicity, oxidative stress, and glial scar formation (Anwar et al., 2016; Hellenbrand et al., 2021). On their own, both MSCs and TIIA have been reported to exert partial but meaningful benefits in SCI repair (Shen et al., 2025). When administered alone, TIIA provides neuroprotection primarily through its anti-inflammatory and antioxidant properties (Shen et al., 2025). TIIA has been shown to suppress neuronal apoptosis, reduce lesion cavity size, and modulate microglial behavior by shifting polarization away from the pro-inflammatory M1 phenotype toward the reparative M2 state (Chen et al., 2020). Mechanistically, this involves inhibition of the NLRP3 inflammasome, resulting in reduced secretion of IL-1β and IL-18, thereby dampening secondary injury cascades. These effects translate into modest but measurable improvements in locomotor recovery, highlighting TIIA’s capacity to reshape the injury microenvironment into one more permissive for neural survival and axonal regrowth.

MSCs, in parallel, have long been considered a promising therapeutic strategy for SCI because of their ability to differentiate into neuronal-like cells, secrete neurotrophic factors, and release extracellular vesicles that modulate local immune responses (Liu et al., 2026; Pang et al., 2023; Xia et al., 2023). Transplanted MSCs can migrate toward lesion sites, reduce glial scarring, and promote limited axonal regeneration (Liu et al., 2026; Pang et al., 2023; Xia et al., 2023). However, the post-injury milieu, characterized by oxidative stress, proinflammatory cytokines, and inhibitory extracellular matrix components, severely restricts MSC survival and neuronal differentiation, curtailing their long-term reparative impact (Ardah et al., 2026; Bao et al., 2025). As a result, MSC transplantation alone tends to produce incomplete functional recovery, with substantial deficits remaining.

The combination of TIIA and MSC therapy, however, generates outcomes that are significantly greater than either intervention in isolation. In the study by Zhang et al. (2018), TIIA not only enhanced locomotor recovery in rats when paired with MSC transplantation but also appeared to influence the lineage fate of transplanted MSCs. Animals receiving both therapies displayed higher expression of neuronal markers such as NeuN, NF200, and Nestin in transplanted cells, with a concomitant reduction in astrocytic differentiation, as reflected by decreased GFAP expression. This shift in differentiation trajectory indicates that TIIA acts not only as a protective agent but also as a lineage-directing cue, biasing MSCs toward neuronal fates that are more beneficial for functional spinal cord repair. Histologically, the combined treatment produced smaller lesion cavities, reduced glial scar formation, and better preservation of spinal cord architecture, while functionally, rats achieved markedly superior locomotor recovery scores compared to groups treated with TIIA or MSCs alone.

A further advance in this line of research comes from the recent work of Shen et al. (2025), which investigated exosomes derived from TIIA-pretreated umbilical cord MSCs (UCMSCs). These TIIA-conditioned exosomes (T-Exos) demonstrated enhanced therapeutic efficacy compared with naïve MSC-derived exosomes. The study revealed that TIIA pretreatment enriched the exosomal cargo with miR-223-5p, a microRNA with strong anti-inflammatory activity. miR-223-5p directly targeted USP8, a deubiquitinase that stabilizes NLRP3, thereby reducing NLRP3 inflammasome activation and shifting microglia toward the anti-inflammatory M2 phenotype. In SCI models, T-Exos not only suppressed the production of proinflammatory cytokines (IL-1β, IL-18) but also alleviated oxidative stress and promoted tissue preservation (Shen et al., 2025; Liu et al., 2026; Li Y. et al., 2024). Functionally, mice treated with T-Exos exhibited significantly better motor recovery, with improved locomotor performance compared to controls or animals treated with unmodified MSC exosomes (Shen et al., 2025). Importantly, inhibition of miR-223-5p abrogated these effects, confirming that the miR-223-5p/USP8/NLRP3 axis was central to the enhanced therapeutic benefits (Shen et al., 2025). This work demonstrates that TIIA does not merely act on MSCs in a supportive capacity but can reprogram their secretome, augmenting the immunomodulatory and neuroprotective potential of their exosomes.

### Cardiovascular disease models

In cardiovascular injury models, particularly MI and myocardial ischemia/reperfusion injury (MI/RI), both TIIA and MSCs exhibit beneficial—but individually limited—therapeutic effects (Othman et al., 2026; Jiang et al., 2018; Hashmi and Al-Salam, 2015). MSCs confer protection largely through paracrine signaling, anti-apoptotic activity, and exosome-mediated modulation of inflammation and angiogenesis (Chen et al., 2016; Zhou et al., 2019). However, their clinical efficacy is restricted by poor survival, limited engraftment, and diminished potency within the ischemic myocardium. TIIA, acting independently, provides anti-inflammatory, antioxidant, and anti-apoptotic effects that reduce tissue injury but cannot fully restore myocardial structure or function.

A pivotal study by Tong et al. (Jiang et al., 2019) demonstrated that TIIA significantly enhances MSC recruitment to ischemic myocardium by activating the SDF-1/CXCR4 axis. *In vitro*, TIIA increased MSC migration by approximately 3.7-fold, an effect abolished by CXCR4 inhibition, confirming the centrality of this chemokine pathway in MSC homing.

Beyond effects on MSC migration, TIIA substantially enhances the therapeutic quality of MSC-derived exosomes. In MI/RI models, TIIA-preconditioned MSC exosomes (TSA-MSCexo), as reported by Li et al. (Du et al., 2025), significantly improved cardiac function, reduced infarct size, promoted angiogenesis, and moderated inflammatory responses. Mechanistically, miR-223-5p enriched within TSA-MSCexo directly targets CCR2, suppressing monocyte infiltration and limiting inflammatory injury while simultaneously enhancing angiogenic signaling. These combined actions promote vascular regeneration and preserve myocardial tissue.

Together, TIIA primes MSCs for improved survival, migration, and paracrine signaling, while MSCs supply trophic factors and bioactive exosomes that regulate apoptosis, inflammation, and angiogenesis. This dual mechanism produces robust synergy, yielding myocardial repair outcomes that exceed those achieved by either agent alone. Collectively, these findings highlight TIIA-preconditioned MSC strategies as a promising therapeutic avenue for ischemic heart disease (Jiang et al., 2019; Du et al., 2025).

## Musculoskeletal and regenerative models

### Bone and fracture healing

In osteoporotic fracture models, TIIA demonstrates pronounced benefits both alone and in combination with MSC-based interventions. Osteoporotic bone is characterized by heightened oxidative stress and a compromised regenerative environment, leading to delayed fracture healing (Patel and Wairkar, 2023; Liu et al., 2023). TIIA pretreatment of MSCs activates Nrf2-dependent antioxidant signaling, reduces intracellular reactive oxygen species (ROS), and protects cells from apoptosis. This preservation of cell viability maintains MSC osteogenic potential, enabling robust differentiation despite oxidative challenges (Li Y. et al., 2024).

*In vivo*, TIIA administration enhances callus mineralization, increases bone mineral density, and improves the biomechanical strength of regenerated bone. When delivered via injectable hydrogels, TIIA further amplifies these effects. The hydrogel provides sustained, localized release of TIIA, improving MSC survival and osteogenic function while creating a pro-regenerative microenvironment (Li Y. et al., 2024). This leads to improved callus formation, enhanced trabecular architecture, and superior mechanical integrity of healed bone.

Thus, TIIA supports bone repair through a dual mechanism: as a standalone agent it confers cytoprotection and stimulates osteogenesis, and in combination with MSCs it produces synergistic enhancement of fracture regeneration (Li Y. et al., 2024).

### Skeletal muscle regeneration

TIIA also exerts potent effects on skeletal muscle regeneration, acting directly on muscle stem cells (MuSCs). Lu et al. (2016) demonstrated that TIIA enhances MuSC proliferation and differentiation, strengthening endogenous regenerative responses following injury. TIIA treatment increases cell cycle entry and upregulates proliferative markers, while promoting myogenic differentiation as evidenced by MyHC expression and elevated levels of myogenic regulatory factors (Lu et al., 2016).

Mechanistically, these effects rely on distinct phases of MAPK and Akt pathway activation. MAPK signaling is preferentially engaged during proliferation, while Akt signaling dominates the differentiation phase. Pharmacological inhibition of these pathways (U0126 for MAPK and Akti-1/2 for Akt) eliminates TIIA’s effects, confirming pathway specificity (Lu et al., 2016).

Functionally, these molecular changes translate to improved myofiber regeneration, enhanced muscle force, and better running performance following injury. Although studies combining TIIA with MuSC transplantation or biomaterial scaffolds are limited, the compound’s dual protective and stimulatory properties suggest potential synergy similar to bone regeneration. TIIA is expected to reduce oxidative and inflammatory stress, improving survival of transplanted MuSCs while simultaneously activating endogenous MuSC pools (Lu et al., 2016).

Collectively, TIIA acts as a “dual-action” modulator in musculoskeletal regeneration: it protects stem cells against stress while stimulating their proliferative and differentiative capacity, yielding superior tissue repair outcomes when used alone or in combination with regenerative therapies.

## Hepatic and reproductive regeneration

### Liver injury and fibrosis

In hepatic injury models, TIIA serves as a potent activator of endogenous liver regeneration (Xu L. et al., 2022; Qi et al., 2010). Independently, TIIA promotes hepatic stem/progenitor cell proliferation and differentiation, improves hepatic architecture, and mitigates fibrosis (Yang et al., 2020). In cirrhotic rat models induced by carbon tetrachloride or alcohol, TIIA reduces collagen deposition, preserves lobular structure, and normalizes biochemical markers including albumin and bilirubin, thereby improving ALBI and MELD scores (Shu et al., 2016). Histologically, TIIA stimulates expansion of EpCAM- and OV-6-positive progenitor cells and promotes their differentiation into albumin- and CK-18-positive hepatocytes. These results indicate that TIIA not only suppresses fibrotic remodeling but also activates lineage commitment pathways that facilitate hepatic regeneration in hostile microenvironments.

### Reproductive regeneration and intrauterine adhesions

A similar pattern emerges in reproductive system repair, particularly in intrauterine adhesions (IUA), a major cause of infertility. TIIA alone exerts significant anti-fibrotic and pro-regenerative effects. In rat IUA models (Xu W. et al., 2022), TIIA reduces fibrosis, increases endometrial thickness, elevates estrogen (E2) levels, and restores regenerative capacity. These effects involve upregulation of miR-29a, which regulates extracellular matrix remodeling, and suppression of profibrotic TGF-β1/Smad3 signaling (Xu W. et al., 2022). However, despite these benefits, TIIA alone cannot fully reconstruct glandular architecture or restore complete fertility potential.

MSCs, including decidual MSCs, provide complementary benefits. dMSCs are naturally adapted to the uterine niche, capable of differentiating into endometrial-like cells and secreting paracrine factors that promote angiogenesis and stromal remodeling. In IUA models, dMSCs alone partially restore endometrial thickness, reduce fibrosis, increase gland number, and enhance E2 levels (Xu W. et al., 2022). However, as in other tissues, their long-term efficacy is limited by poor survival and the fibrotic microenvironment.

The combination of TIIA and dMSCs yields superior outcomes. Xu et al. (Song et al., 2025) reported that co-administration significantly reduced fibrosis area, restored endometrial thickness and glandular density to near-normal levels, and enhanced angiogenic balance. Mechanistically, TIIA-dMSC therapy modulated miR-29a to suppress TGF-β1/Smad3 signaling, improved systemic E2 levels, and regulated MMP-9 expression to optimize extracellular matrix remodeling. TIIA reshaped the uterine microenvironment into an anti-fibrotic, pro-regenerative niche, while dMSCs provided the cellular machinery for structural and functional repair.

Taken together, these results illustrate that TIIA acts as both a pharmacologic activator of endogenous repair and a synergistic enhancer of MSC-based therapies. In hepatic and reproductive systems, TIIA reduces fibrosis, supports stem cell survival, and promotes tissue-specific differentiation. When combined with MSCs, it overcomes microenvironmental barriers that typically limit cell-based therapy, enabling robust architectural and functional regeneration.

## Discussion

The present review highlights the emerging therapeutic synergy between TIIA and MSCs across multiple disease models, including neurodegenerative, cardiovascular, musculoskeletal, hepatic, and reproductive disorders (Table 1). While both TIIA and MSCs independently exhibit regenerative and protective properties (Santoro et al., 2025; Han et al., 2025), accumulating evidence demonstrates that their combined application substantially enhances therapeutic efficacy by addressing key limitations inherent to each modality (Song et al., 2025).

Another key consideration is the pronounced methodological heterogeneity across published studies. Variations in the MSC tissue source, TIIA concentration, pretreatment duration, route of administration, and disease models make direct comparisons difficult. While BM-MSCs are the most frequently investigated cell population, exosomes derived from UC-MSCs have recently demonstrated particularly strong immunomodulatory activity (Yi et al., 2025), whereas decidual MSCs seem beneficial for reproductive applications (Yu et al., 2025). Likewise, local biomaterial-assisted delivery may yield greater therapeutic advantages than systemic administration by enhancing both MSC retention and prolonged TIIA exposure. Taken together, these findings indicate that treatment efficacy depends not only on TIIA itself but also on the appropriate selection of the stem-cell source and the delivery platform.

A central challenge in stem cell–based therapy is the low survival and limited engraftment of transplanted cells within hostile pathological microenvironments characterized by oxidative stress, inflammation, hypoxia, and fibrosis (Almalki and Agrawal, 2016; Meirelles Lda and Nardi, 2009; Dabrowska et al., 2020). TIIA appears to mitigate these barriers through its well-documented antioxidant, anti-inflammatory, and anti-apoptotic properties (Du et al., 2025); Tanshinones, Critical Pharmacological Components in Salvia miltiorrhiza). Activation of signaling pathways such as Nrf2, MAPK, Akt, and SDF-1/CXCR4 plays a critical role in improving MSC viability, migration, and paracrine activity (Li Y. et al., 2024; Tong et al., 2011; Lu et al., 2016). By reshaping the tissue microenvironment and directly priming MSCs, TIIA enhances stem cell resilience and functional integration following transplantation.

Another important mechanism underlying this synergy is the enhancement of MSC-derived paracrine signaling, particularly through extracellular vesicles and exosomes. Several studies indicate that TIIA preconditioning alters the molecular cargo of MSC exosomes, enriching regulatory microRNAs such as miR-223-5p, which target inflammatory and immune pathways including CCR2 and USP8-mediated NLRP3 inflammasome activation (Shen et al., 2025). These modifications promote macrophage polarization toward anti-inflammatory phenotypes, reduce immune cell infiltration, and support angiogenesis and tissue remodeling (Othman et al., 2026; Chen et al., 2016; Zhou et al., 2019). Consequently, TIIA not only improves MSC survival but also amplifies the therapeutic potency of MSC secretomes (Ding et al., 2010).

The benefits of this combined strategy are evident across diverse organ systems. In neurodegenerative diseases, including AD and vascular dementia, TIIA–MSC therapy synergistically suppresses pro-inflammatory cytokines, reduces amyloid-β accumulation, and enhances neuronal survival and synaptic function. In spinal cord injury, TIIA facilitates neuronal differentiation of MSCs, decreases glial scar formation, and improves locomotor recovery (Shen et al., 2025), functioning as both a neuroprotective agent and a lineage-directing cue (Ardah et al., 2026; Lu et al., 2016).

In cardiovascular disease, particularly MI and ischemia/reperfusion injury, TIIA enhances MSC recruitment via the SDF-1/CXCR4 chemokine axis, thereby improving homing to injured myocardium (Tong et al., 2011; Shen et al., 2025). Additionally, exosomes derived from TIIA-preconditioned MSCs demonstrate enhanced anti-inflammatory and angiogenic capabilities, significantly improving cardiac function and reducing infarct size (Jiang et al., 2018; Shen et al., 2025). These findings underscore the potential of pharmacologically optimized MSC therapies in overcoming the limited retention and functionality of stem cells in ischemic tissues (Othman et al., 2026).

Similarly, in musculoskeletal regeneration, TIIA protects MSCs against oxidative stress and preserves their osteogenic capacity through activation of Nrf2-mediated antioxidant pathways Pharmacological Effects and Mechanisms of TIIA in Bone Injury Repair (Li Y. et al., 2024). This effect promotes fracture healing, enhances bone mineral density, and improves biomechanical strength (Patel and Wairkar, 2023; Lu et al., 2026). TIIA also directly stimulates MuSCs, activating MAPK and Akt signaling to promote myogenic proliferation and differentiation (Lu et al., 2016). These dual actions suggest that TIIA can simultaneously support transplanted stem cells and activate endogenous regenerative pathways.

In hepatic and reproductive systems, the combination of TIIA and MSCs demonstrates notable anti-fibrotic and regenerative effects. In liver injury models, TIIA promotes hepatic progenitor cell expansion and differentiation while reducing collagen deposition (Yi et al., 2025). In intrauterine adhesion models, TIIA synergizes with decidual MSCs to suppress TGF-β1/Smad3 signaling, enhance miR-29a expression, and restore endometrial thickness and glandular architecture (Xu W. et al., 2022). These findings indicate that TIIA can reshape fibrotic microenvironments into conditions conducive to stem cell–mediated regeneration (Bao et al., 2025).

## Integrated molecular mechanisms underlying TIIA–MSC synergy

Although signaling pathways have been characterized across a range of disease models, accumulating evidence indicates that they operate as an interconnected regulatory network instead of acting independently. Nrf2 is one of the upstream antioxidant regulators activated by TIIA; it decreases oxidative stress and helps maintain MSC viability, especially during osteogenic differentiation (Cheng et al., 2024). By improving redox homeostasis, TIIA then supports the activation of survival pathways such as MAPK and Akt, which in turn control MSC proliferation, migration, and differentiation (Cheng et al., 2024; Liu et al., 2023).

In inflammatory settings, TIIA additionally tunes innate immune signaling through several coordinated mechanisms. By activating TREM2, it suppresses excessive microglial activation and increases phagocytic activity in neuroinflammatory models (Li Y. et al., 2024). In parallel, exosomal miR-223-5p acts on both CCR2 and USP8, thereby preventing NLRP3 inflammasome activation and lowering inflammatory responses in macrophages and microglia (Shen et al., 2025). Similarly, miR-29a inhibits TGF-β1/Smad3 signaling, restricting extracellular matrix deposition and fibrosis during endometrial repair (Xu W. et al., 2022). Collectively, these processes redirect damaged tissues from a pro-inflammatory, pro-fibrotic condition toward a regenerative microenvironment.

A further emerging regulator is AMPK, which has been shown to mediate multiple pharmacological effects of TIIA, including mitochondrial protection, oxidative stress mitigation, and metabolic adaptation in cardiovascular and musculoskeletal tissues (Du et al., 2025). Even though direct evidence connecting AMPK activation to TIIA-preconditioned MSCs is still limited, cross-talk among AMPK, Nrf2, Akt, and MAPK signaling likely helps explain enhanced cellular survival and regenerative potential. Future systems biology studies that integrate transcriptomics, proteomics, and metabolomics will be instrumental in more fully characterizing these linked signaling networks.

## Translational challenges and clinical perspectives

Despite the encouraging preclinical evidence demonstrating synergistic therapeutic effects of TIIA and MSCs, several important challenges must be overcome before this combinatorial strategy can be translated into routine clinical practice. A major limitation relates to the pharmacokinetic properties of TIIA. Owing to its lipophilic nature, TIIA exhibits poor aqueous solubility, relatively low oral bioavailability, rapid hepatic metabolism, and a short systemic half-life, all of which may limit its therapeutic efficacy when administered systemically (Jiang et al., 2019; Du et al., 2025). Consequently, considerable interest has focused on developing advanced drug-delivery systems, including nanoparticles, liposomes, injectable hydrogels, microspheres, and scaffold-based sustained-release platforms, to improve TIIA stability, prolong local retention, enhance tissue targeting, and reduce systemic toxicity (Hu et al., 2025; Du et al., 2025; Lu et al., 2016). Future studies should systematically compare these formulations to determine the optimal delivery strategy for combination therapy with MSCs.

Another important translational issue is the lack of standardized treatment protocols. Current preclinical studies employ considerable variability in TIIA dosage, pretreatment duration, timing of administration, MSC source, cell dose, and delivery route, making direct comparisons difficult (Wong et al., 2026; Han et al., 2025; Song et al., 2025; Pînzariu et al., 2025). For example, TIIA has been administered as systemic injections, local hydrogel formulations, nanoparticle-encapsulated preparations, or as a preconditioning agent during MSC culture, while MSCs have been derived from bone marrow, adipose tissue, umbilical cord, decidua, and other tissues (Song et al., 2025; Orbay et al., 2012; Cheng et al., 2024; Saparov et al., 2016; Kahrizi et al., 2023). These differences may substantially influence cell survival, migration, differentiation, paracrine activity, and overall therapeutic efficacy. Therefore, future investigations should establish standardized protocols that define optimal TIIA concentrations, pretreatment duration, cell dosage, administration schedules, and disease-specific delivery routes to improve reproducibility and facilitate multicenter clinical trials (Wong et al., 2026; Tu et al., 2026; Han et al., 2025; Pînzariu et al., 2025) (Wong et al., 2026; Tu et al., 2026; Han et al., 2025; Pînzariu et al., 2025).

Long-term safety also remains insufficiently characterized. Although both MSC- and TIIA-based therapies have generally demonstrated favorable safety profiles in preclinical studies (Wong et al., 2026; Du et al., 2025; Han et al., 2025), robust long-term evaluations of combination therapy are still lacking. Future investigations should include comprehensive assessments of biodistribution, persistence of transplanted cells and extracellular vesicles, immunogenicity, repeated-dose toxicity, potential tumorigenicity, ectopic tissue formation, and pharmacokinetic interactions between TIIA and transplanted MSCs (Wong et al., 2026; Han et al., 2025; Pînzariu et al., 2025; Garza Treviño et al., 2024) (2,7,13,25). Such studies are particularly important because pharmacological enhancement of MSC function could potentially alter stem-cell behavior, exosome composition, or host immune responses following transplantation.

The rapid development of MSC-derived extracellular vesicles and exosomes also introduces new regulatory and manufacturing challenges. Although exosomes derived from TIIA-preconditioned MSCs have demonstrated enhanced regenerative and immunomodulatory activity in several disease models (Li et al., 2023; Shen et al., 2025), their clinical translation will require rigorous quality-control standards. Critical factors include donor selection, MSC tissue source, culture conditions, cell passage number, exosome isolation and purification methods, physicochemical characterization, potency assays, sterility testing, storage stability, and batch-to-batch consistency (Song et al., 2025; Zhan et al., 2020; Ma et al., 2020; Dabrowska et al., 2020; Han et al., 2022; Othman et al., 2026). Establishing Good Manufacturing Practice (GMP)-compliant production protocols and internationally accepted release criteria will be essential to ensure reproducibility, product safety, and regulatory approval for future clinical applications (Kaiser et al., 2022; Gao et al., 2020).

Finally, successful clinical translation will require well-designed randomized clinical trials that evaluate not only therapeutic efficacy but also optimal patient selection, treatment timing, dosing regimens, and long-term follow-up (Wong et al., 2026; Tu et al., 2026; Han et al., 2025; Pînzariu et al., 2025). Integrating biomaterials, controlled drug-delivery systems, and cell-free therapeutic approaches using TIIA-preconditioned MSC-derived exosomes may further improve cell retention, enhance local therapeutic effects, and reduce the risks associated with live-cell transplantation (Song et al., 2025; Liu et al., 2026; Li Y. et al., 2024; Lu et al., 2026; Kaiser et al., 2022). Moreover, advances in multi-omics technologies and precision medicine approaches may facilitate biomarker discovery and enable personalized regenerative therapies tailored to individual disease characteristics. Collectively, overcoming these pharmacological, manufacturing, regulatory, and clinical challenges will be essential for realizing the full therapeutic potential of TIIA-enhanced MSC therapies in regenerative medicine.

## Limitation

Despite promising preclinical findings, several key translational obstacles still need to be addressed before TIIA-enhanced MSC therapy can be adopted in clinical practice. First, almost all existing evidence comes from rodent models, with only minimal validation in large-animal models or human clinical trials. As a result, species-specific differences in immune responses, stem-cell biology, and pharmacokinetics may constrain extrapolation to patients.

Second, substantial heterogeneity is present across MSC sources, isolation methods, passage numbers, culture conditions, and TIIA pretreatment protocols. This variability makes it difficult to compare results across studies and underscores the need for standardized manufacturing procedures in line with GMP guidelines.

Third, the pharmacokinetic properties of TIIA are not yet fully defined in the setting of regenerative medicine. Native TIIA shows poor aqueous solubility, limited oral bioavailability, rapid metabolism, and a relatively brief systemic half-life, all of which could diminish therapeutic effectiveness. More advanced delivery platforms, including nanoparticles, liposomes, hydrogels, and sustained-release biomaterials, may increase local drug retention and strengthen synergy with MSC transplantation (Yi et al., 2025; Yu et al., 2025; Liu et al., 2023).

Fourth, although MSC-derived exosomes offer a compelling cell-free therapeutic alternative, major technical challenges persist with respect to large-scale manufacturing, purification, characterization, storage stability, potency assessment, and batch-to-batch quality control. Developing standardized manufacturing protocols will be critical prior to clinical translation.

Finally, long-term safety has not been sufficiently characterized. While both TIIA and MSCs show favorable safety profiles on their own, potential effects of extended TIIA exposure on transplanted cells, including changes in biodistribution, immune modulation, ectopic differentiation, or tumor-promoting outcomes, must be evaluated through systematic long-term preclinical and clinical studies.

## Future directions

Despite promising preclinical findings, several key areas require additional investigation to support the clinical translation of TIIA-enhanced MSC therapy. First, because most existing studies have been carried out in rodent models, there is a clear need for rigorously designed large-animal experiments and early-phase clinical trials to assess the safety, efficacy, and most effective therapeutic regimens of TIIA-MSC combinations in humans. Future clinical studies should define standardized dosing schedules, pretreatment duration, administration timing, and delivery routes, while also evaluating the pharmacokinetic and pharmacodynamic interactions between TIIA and transplanted MSCs. Since TIIA has limited aqueous solubility and bioavailability, optimizing drug formulations—such as nanoparticle-, liposome-, and hydrogel-based delivery systems—may further enhance therapeutic efficacy and should be assessed in a systematic manner.

Another key priority is to standardize manufacturing and pretreatment protocols for MSCs. MSCs obtained from bone marrow, adipose tissue, umbilical cord, placenta, and decidua exhibit distinct biological characteristics, immunomodulatory abilities, and regenerative potential, which may affect their responsiveness to TIIA. Accordingly, comparative studies that directly evaluate different MSC sources under identical experimental conditions are necessary to determine the most appropriate cell populations for particular disease indications. In parallel, standardized criteria concerning cell passage number, culture conditions, TIIA concentration, pretreatment duration, and quality-control procedures are needed to enhance reproducibility across laboratories and to support regulatory approval.

Future mechanistic research should also extend beyond individual signaling pathways toward a systems-level understanding of TIIA-mediated MSC regulation. While pathways such as Nrf2, MAPK, Akt, SDF-1/CXCR4, TREM2, AMPK, miR-223-5p/USP8/NLRP3, and miR-29a/TGF-β1/Smad3 have been implicated, their upstream regulators, downstream targets, and possible crosstalk are still not fully understood. Integrative transcriptomic, proteomic, metabolomic, and single-cell sequencing strategies, together with bioinformatics and systems biology analyses, may yield a more comprehensive view of the molecular networks that underlie the enhanced regenerative capacity of TIIA-preconditioned MSCs.

Cell-free therapeutic approaches are also an important avenue for future development. Exosomes obtained from TIIA-preconditioned MSCs have been shown to exhibit improved anti-inflammatory, pro-angiogenic, and regenerative effects, while potentially circumventing multiple drawbacks linked to cell transplantation. Nevertheless, prior to clinical use, standardized procedures for exosome isolation, characterization, potency evaluation, storage, and large-scale production under GMP conditions must be defined to guarantee consistent therapeutic quality and reproducible outcomes.

Alongside this, biomaterial-assisted delivery systems, such as injectable hydrogels, aligned microfibers, biodegradable scaffolds, and tissue-engineered constructs, should be further refined to increase MSC retention, extend localized TIIA release, and strengthen tissue-specific regeneration. Integrating pharmacological preconditioning with biomaterial engineering may provide a practical route to enhance both cell survival and regenerative performance within hostile pathological microenvironments.

Long-term safety is likewise a key concern. Future research should incorporate thorough evaluations of biodistribution, immune responses, genomic stability, ectopic tissue formation, tumorigenicity, and chronic toxicity after repeated administration of TIIA-MSC therapies or their exosomes. Finally, extending studies to additional disease models, including autoimmune, metabolic, chronic inflammatory, and aging-associated conditions, may further widen the therapeutic scope of this combinatorial strategy. The incorporation of precision medicine strategies, including biomarker-guided patient selection and artificial intelligence-supported multi-omics analyses, may ultimately allow personalized TIIA-MSC therapies designed for the specific disease features and regenerative needs of individual patients.

## Conclusion

TIIA and MSCs represent two complementary therapeutic approaches in regenerative medicine. While MSCs provide cellular and paracrine mechanisms for tissue repair, their efficacy is often limited by poor survival, low engraftment, and hostile pathological environments. TIIA, a bioactive compound with potent antioxidant, anti-inflammatory, and anti-fibrotic properties, effectively addresses many of these limitations.

Evidence from multiple disease models demonstrates that TIIA significantly enhances MSC survival, migration, differentiation, and paracrine activity, thereby amplifying their regenerative capacity. Mechanistically, this synergy involves activation of protective signaling pathways, modulation of inflammatory responses, and enhancement of MSC-derived exosome function. These combined effects lead to improved outcomes in neurological repair, cardiac regeneration, musculoskeletal healing, hepatic recovery, and reproductive tissue restoration.

Importantly, the integration of pharmacological agents such as TIIA with stem cell therapies represents a promising strategy to optimize regenerative medicine approaches. By improving the biological performance of transplanted cells and reshaping damaged tissue microenvironments, TIIA-MSC therapies may overcome several barriers that currently limit clinical translation.

Future research should focus on well-designed clinical studies, standardized therapeutic protocols, and deeper mechanistic investigations to fully elucidate the therapeutic potential of this combination. With continued development, TIIA-enhanced MSC therapy may emerge as a versatile and effective treatment platform for a wide range of degenerative and inflammatory diseases.
